# Vascular surgical stretch injury leads to activation of P2X7 receptors and impaired endothelial function

**DOI:** 10.1371/journal.pone.0188069

**Published:** 2017-11-14

**Authors:** Padmini Komalavilas, Weifeng Luo, Christy M. Guth, Olukemi Jolayemi, Rachel I. Bartelson, Joyce Cheung-Flynn, Colleen M. Brophy

**Affiliations:** 1 Vanderbilt University Medical Center, Department of Surgery, Nashville, TN, United States of America; 2 VA Tennessee Valley Healthcare System, Nashville, TN, United States of America; Universite Paris-Sud, FRANCE

## Abstract

A viable vascular endothelial layer prevents vasomotor dysfunction, thrombosis, inflammation, and intimal hyperplasia. Injury to the endothelium occurs during harvest and “back table” preparation of human saphenous vein prior to implantation as an arterial bypass conduit. A subfailure overstretch model of rat aorta was used to show that subfailure stretch injury of vascular tissue leads to impaired endothelial-dependent relaxation. Stretch-induced impaired relaxation was mitigated by treatment with purinergic P2X7 receptor (P2X7R) inhibitors, brilliant blue FCF (FCF) and A740003, or apyrase, an enzyme that catalyzes the hydrolysis of ATP. Alternatively, treatment of rat aorta with exogenous ATP or 2’(3’)-O-(4-Benzoyl benzoyl)-ATP (BzATP) also impaired endothelial-dependent relaxation. Treatment of human saphenous vein endothelial cells (HSVEC) with exogenous ATP led to reduced nitric oxide production which was associated with increased phosphorylation of the stress activated protein kinase, p38 MAPK. ATP- stimulated p38 MAPK phosphorylation of HSVEC was inhibited by FCF and SB203580. Moreover, ATP inhibition of nitric oxide production in HSVEC was prevented by FCF, SB203580, L-arginine supplementation and arginase inhibition. Finally, L-arginine supplementation and arginase inhibition restored endothelial dependent relaxation after stretch injury of rat aorta. These results suggest that vascular stretch injury leads to ATP release, activation of P2X7R and p38 MAPK resulting in endothelial dysfunction due to arginase activation. Endothelial function can be restored in both ATP treated HSVEC and intact stretch injured rat aorta by P2X7 receptor inhibition with FCF or L-arginine supplementation, implicating straightforward therapeutic options for treatment of surgical vascular injury.

## Introduction

Human saphenous vein (HSV) is harvested from the leg and transplanted as a bypass graft into the coronary or peripheral circulation. Vein graft failure rates remain high (45% and 39%, respectively, at 12–18 months per the PREVENT trials [1, 2]). A major source of vein graft injury is during harvest and preparation prior to implantation, with the fragile endothelial monolayer being the most susceptible to injury [3–8]. A functional endothelial layer is important for prevention of vasomotor dysfunction, thrombosis, inflammation, and intimal hyperplasia [9].

Endoscopic vein harvest has been widely adopted to reduce the incidence of leg wound complications. However, analysis of the PREVENT IV data demonstrated that endoscopic vein harvest is associated with increased vein graft failure [10]. This may be due to the increased injury that occurs during endoscopic harvest, as it requires greater traction on the HSV.

To understand surgical traction injury, a rat aorta (RA) model of subfailure overstretch injury was developed [11]. Subfailure overstretch represents the length of stretch at the level of a haptic endpoint (tactile feedback due to tension from the vessel leads to a discernable endpoint). This length is above the *in vivo* length but well below the length at which the tissue fails (1.5–2 times of the *ex vivo* length) [4, 11]. This level of stretch injury is described as subfailure overstretch injury to indicate that it is a pathologic stretch injury, but does not lead to disruption of the vessel. Subfailure overstretch injury is characterized by impaired vascular smooth muscle contraction [11]. Similar traction stretch injury leads to impaired vasomotor function of porcine saphenous vein (PSV), [4, 12, 13] and HSV [4, 6] suggesting that subfailure stretch injury leads to impaired function of both arterial and venous tissues.

In addition to stretch injury, many surgeons mark HSV off-label with a surgical skin marker (SSM) to preserve orientation during implantation. SSMs contain isopropyl alcohol as the solvent and gentian violet dye, both of which are cytotoxic and lead to decreased viability of the conduit [3]. To limit injury during marking, a non-toxic, water soluble food dye, brilliant blue FCF (FCF) was identified [8]. FCF restored functional responses after stretch injury of PSV [13] and endoscopically harvested HSV [14]. FCF is a P2X7 receptor (P2X7R) antagonist [13–15]. P2X7R are activated by sustained exposure to high concentrations of ATP. P2X7R activation results in the formation of large membrane pores, influx of calcium and activation of caspases, and ultimately apoptosis [16]. Treatment with P2X7R antagonists has been shown to ameliorate spinal cord injury [17] and various inflammatory and neurological disorders [18] in animal models. A recently developed rat aorta (RA) model of subfailure overstretch injury showed that vascular stretch injury led to impaired contractile function that also was partially restored with inhibitors of P2X7R [11, 19]. Since subfailure overstretch injury was associated with release of ATP in rat aorta [20] and FCF, a P2X7R inhibitor restored vasomotor dysfunction after stretch injury, P2X7R activation is postulated to play a role in stretch injury of vascular tissue.

While the “**response to injury***” hypothesis* has been described as underlying cause of vascular graft failure, there is very little information about the molecular mediator of this process. The hypothesis of this study was that vascular stretch injury activates P2X7R leading to activation of a signaling cascade that results in **endothelial dysfunction**. Endothelial dysfunction is central to vascular graft pathology.

## Materials and methods

All chemicals were purchased from Sigma-Aldrich Co. (St. Louis, MO), unless otherwise indicated. SB 203580, N^ω^ –hydroxy-nor-Arginine and carbachol were obtained from EMD Millipore Corp., Billerica, MA. Plasma-Lyte was purchased from Baxter Corporation (Deerfield, IL). Urea and CHAPS (3-[3-Cholamidopropyl) dimethylammonio-1-propanesulfonate) were from Research Organics Inc. (Cleveland, OH).

### Injury models using rat aorta:

RA was collected from euthanized female, 250–300g, Sprague Dawley rats. This study was carried out in strict accordance with the recommendations in the Guide for the Care and Use of Laboratory Animals of the National Institute of Health. The protocol was approved by the Institutional Animal Care and Use Committee of the Vanderbilt University Medical Center. Immediately after euthanasia by CO_2_ exposure, the aorta was isolated via a midline incision, placed in heparinized Plasma-Lyte (HP, 10 units heparin/mL Plasma-Lyte) and transported to the laboratory for immediate testing.

RA was manually stretched longitudinally to 200% the *ex vivo* length for 10 seconds and repeated twice as described earlier [11]. Stretched RA was cut into 2–3 mm segments and incubated for 1 hour at room temperature in HP with or without apyrase (4 units/mL), P2X7R antagonists, brilliant blue FCF (FCF, 100 μM, VasoPrep, Morristown, NJ) or A740003 (100 μM), the p38 MAP kinase inhibitor, SB 203580 (SB, 20 μM), arginase inhibitor N^ω^ –hydroxy-nor-Arginine (NOHA, 50 μM) or L arginine (2 mM). For ATP or BzATP treatment, unstretched RA was cut into 2–3mm segments and treated with ATP (2,5,10, 20, and 25 mM) or BzATP (0.5, 1.0, 2.0 and 5.0 mM) in HP for 1 hour at room temperature. RA was also treated with 20 mM ATP or with ATP in the presence of 20 μM PD 98059 [2-(2-Amino-3-methoxyphenyl)-4H-1-benzopyran-4-one] for 1 hr at room temperature.

### Physiologic responses

Rings of RA (stretched or treated with ATP, BzATP, or ATP+PD 98059) were suspended in a muscle bath containing a bicarbonate buffer (120 mM sodium chloride, 4.7 mM potassium chloride, 1.0 mM magnesium sulfate, 1.0 mM monosodium phosphate, 10 mM glucose, 1.5 mM calcium chloride, and 25 mM sodium bicarbonate, pH 7.4), equilibrated with 95% O_2_ / 5% CO_2_ at 37°C for 1 hr. Rings were manually stretched to 3 g of tension, and maintained at a resting tension of 1 g for an additional 1 hr. This produced the maximal force-tension relationship as previously described [14, 21, 22]. After equilibration, the rings were contracted with 110 mM potassium chloride (with equimolar replacement of sodium chloride in bicarbonate buffer) to determine smooth muscle functional viability. To determine endothelial-dependent relaxation, the tissue was then contracted with phenylephrine (PE, 0.5 μM) and relaxed with carbachol (CCH, 0.5 μM), an acetylcholine analogue [23]. Force measurements were obtained using the Radnoti force transducer (model 159901A,Radnoti LLC, Monrovia, CA) interfaced with a PowerLab data acquisition system and Chart software (AD Instruments Inc., Colorado Springs, CO) and was converted to stress by adjusting to the length and weight of the tissue. Contractile response was defined as stress ([10^5^ Newtons (N)/m^2^] = force (g) x 0.0987/area, where area is equal to the wet weight [(mg)/length (mm at maximal length)] divided by 1.055, which was calculated using the force (g) generated by the tissue. Percent relaxation was calculated as a change in stress compared to the maximal PE-induced contraction which was set as 100% as described earlier [14].

### Human saphenous vein endothelial cell culture

Human Saphenous Vein Endothelial Cells obtained commercially (HSVEC, C-12231, PromoCell, Heidelberg, Germany) were grown in Endothelial Cell Growth Medium (C-22010, PromoCell), maintained in a 37°C and 5% CO_2_ incubator and were passaged at 80% confluence. Cells between passages 2 and 5 were used in the experiments.

### Nitric oxide measurement

HSVEC were plated in 60-mm dishes and grew to confluency of >70%. Cells were either left untreated or treated with ATP (2 mM), L-N^G^-Nitroarginine methyl ester (L-NAME, (100 μ), ATP (2 mM) plus FCF (100 μM), ATP (2 mM) plus L arginine (2 mM), or ATP (2 mM) plus N^ω^ –hydroxy-nor-Arginine (NOHA, 10 μM), in growth medium diluted to 50% with basal medium for 1 hr. At the end of the treatment, cells were stimulated with CCH (1 μM) for 10 min and nitric oxide generated was measured as nitrite using the nitric oxide assay kit (ab65327, Abcam, Cambridge, MA) per the manufacturer’s protocol. Relative percent of NO generated was calculated. NO generated with CCH stimulation was set as 100%.

### Immunoblotting

HSVEC were plated in 60 mm dishes and cells at 70% confluency were treated with ATP (2 mM, 10, 30, 60 and 120 min), BzATP (0.25 and 0.5 mM, 120 min) or ATP with 10 μM PD 98059 in culture medium diluted to 50% with basal medium for different duration indicated). To examine the effect of inhibitors, cells were pretreated with the inhibitors for 1hr prior to treating with ATP for 30 min. Proteins were extracted using lysis buffer containing 50 mM Tris. Cl pH 7.4, 140 mM NaCl, 1% NP40, 1 mM EDTA, 1 mM EGTA, 0.5% deoxycholic acid with protease and phosphatase inhibitor cocktail. The proteins were separated using SDS-PAGE and transferred onto nitrocellulose membrane, followed by immunoblotting with antibodies against phospho-p38 MAPK Thr180/Tyr182 (9211, Cell Signaling, Beverly, MA), p38 MAPK (9212, Cell Signaling), phospho ERK1/2 (P-p44/42 MAPK, T202/Y204, Cell Signaling). ERK1/2 (Cell Signaling), phospho SAPK/JNK (T183/Y185) and pan SAPK/JNK (Cell Signaling). P2X7R expression was determined by extracting proteins using lysis buffer followed by a urea chaps buffer (8M urea, 10 mM dithiothreitol, 4% CHAPS) from untreated HSVEC and human HEK 293 cells using anti P2X7R antibodies (Alomone Labs, Israel; #APR-008). Specificity was determined by probing the membrane with the same antibodies preabsorbed with the immunogen peptide. Protein-antibody complexes were visualized and quantified using the Odyssey Infrared Imaging System. Phosphorylation was calculated as a ratio of the phosphorylated protein to total protein and was then normalized to the ATP-stimulated control with the value set as 100%.

### Statistical analysis

Data were reported as standard error of the mean (SEM) responses. Paired t-tests or One-way ANOVA analyses with Tukey’s Multiple Comparison test between columns were conducted in order to determine the significance (*P* value) of experiments. *P* value <0.05 was considered statistically significant.

## Results

### Stretch injury results in reversible endothelial dysfunction

Straightforward methods to mitigate preparation-associated injury in saphenous vein graft have been proposed [24], therefore the goal of this work was to isolate harvest-induced stretch injury. A subfailure overstretch RA model has been previously developed that results in smooth muscle dysfunction, reversible by treatment with P2X7R inhibitors [11].

**Fig 1:**
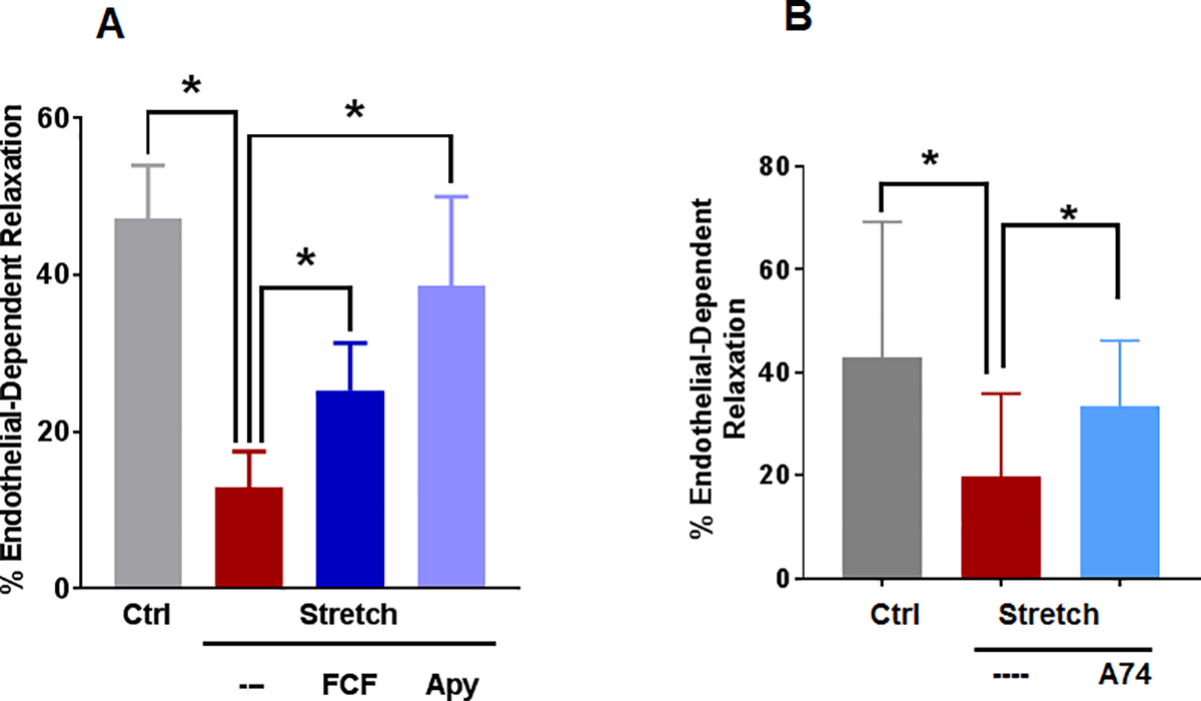
Decreased endothelial function in a subfailure overstretch RA model was restored with apyrase and P2X7R antagonists FCF and A740003. **A.** RA was nonstretched (Ctrl), longitudinally stretched to twice its ex vivo length (Stretch), longitudinally stretched before treatment with FCF (100 μM, Stretch+FCF) or stretched before treatment with apyrase (4units/mL, Stretch+Apy). Vessels were pre-contracted with phenylephrine (0.5 μM) and relaxed with carbachol (0.5 μM), n = 7–15, * p < 0.05 compared to control, (paired t-test). **B**, RA was unstretched (Ctrl) or stretched and incubated with A740003 [19, 26] (100 μM, Stretch+A74) and the vessels were pre-contracted with phenylephrine (0.5 μM) and treated with carbachol (0.5 μM). n = 12 segments from different rats, * p<0.05, (paired t-test).

To determine if stretch injury leads to endothelial dysfunction, RA were subjected to subfailure overstretch as previously described [11], and endothelial-dependent relaxation was determined by pre-contracting the vessels with phenylephrine (PE) followed by the acetylcholine receptor agonist, carbachol (CCH). Subfailure overstretch of RA led to impaired endothelial-dependent relaxation (13 ± 4% vs 42 ± 9%, n = 15, p < 0.05, Fig 1A) which was prevented by treatment with the P2X7R inhibitors, FCF (13 ± 4% vs 25 ± 6%, n = 7, p < 0.05) and A740003 [25] (20 ± 4% vs 33 ±4%, n = 12, p < 0.05, Fig 1B). Treatment of stretched RA with apyrase, which hydrolyzes ATP, abolished endothelial impairment (13 ± 4% vs 39 ± 11%, n = 8, p < 0.05), suggesting that this injury is at least in part due to extracellular ATP.

**Fig 2:**
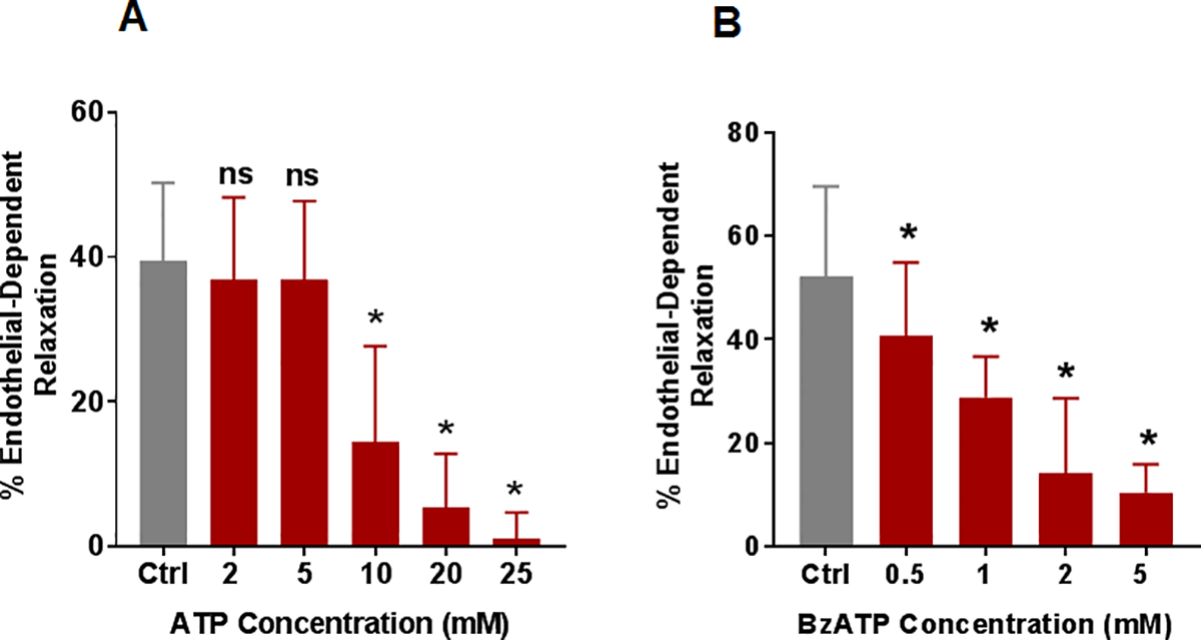
eATP stimulation of RA decreased endothelial function. **A.** RA rings were treated with eATP (2,5,10, 20 and 25 mM) for 1hour, suspended in the muscle bath, pre-contracted with phenylephrine (0.5 μM) and treated with carbachol (0.5 μM), n = 5–10, * p < 0.05 compared to control, ns = not significant compared to control. **B**. RA rings were suspended in a muscle bath and either untreated or treated with 2’(3’)-O-(Benzoylbenzoyl) adenosine 5’–triphosphate (BzATP, 0.5, 1, 2, and 5 mM) for 1 hr, and the vessels were pre-contracted with phenylephrine (0.5 μM) and treated with carbachol (0.5 μM). n = 4–7 segments from different rats, * p<0.05 compared to control (paired t-test).

To confirm the role of extracellular ATP in impaired endothelial-dependent relaxation after subfailure overstretch injury, non-stretched RA were treated with exogenous ATP (eATP). eATP dose consistent with intracellular ATP levels [27] (10 mM) significantly decreased endothelial relaxation (15 ± 5%) compared to control (39 ± 3%, n = 10, p < 0.05) (Fig 2A, S1 Fig). To determine the involvement of P2X7R, RA were treated with BzATP, a selective P2X7R agonist. BzATP (0.5, 1,2 and 5.0 mM) significantly reduced endothelial relaxation in a dose-dependent manner (29 ± 3%, 14 ± 6% and 11 ± 3% for 1, 2, and 5 mM, respectively, compared to control, 52 ± 6%, Fig 2B).

### eATP treatment of HSV endothelial cells leads to impaired nitric oxide release

**Fig 3:**
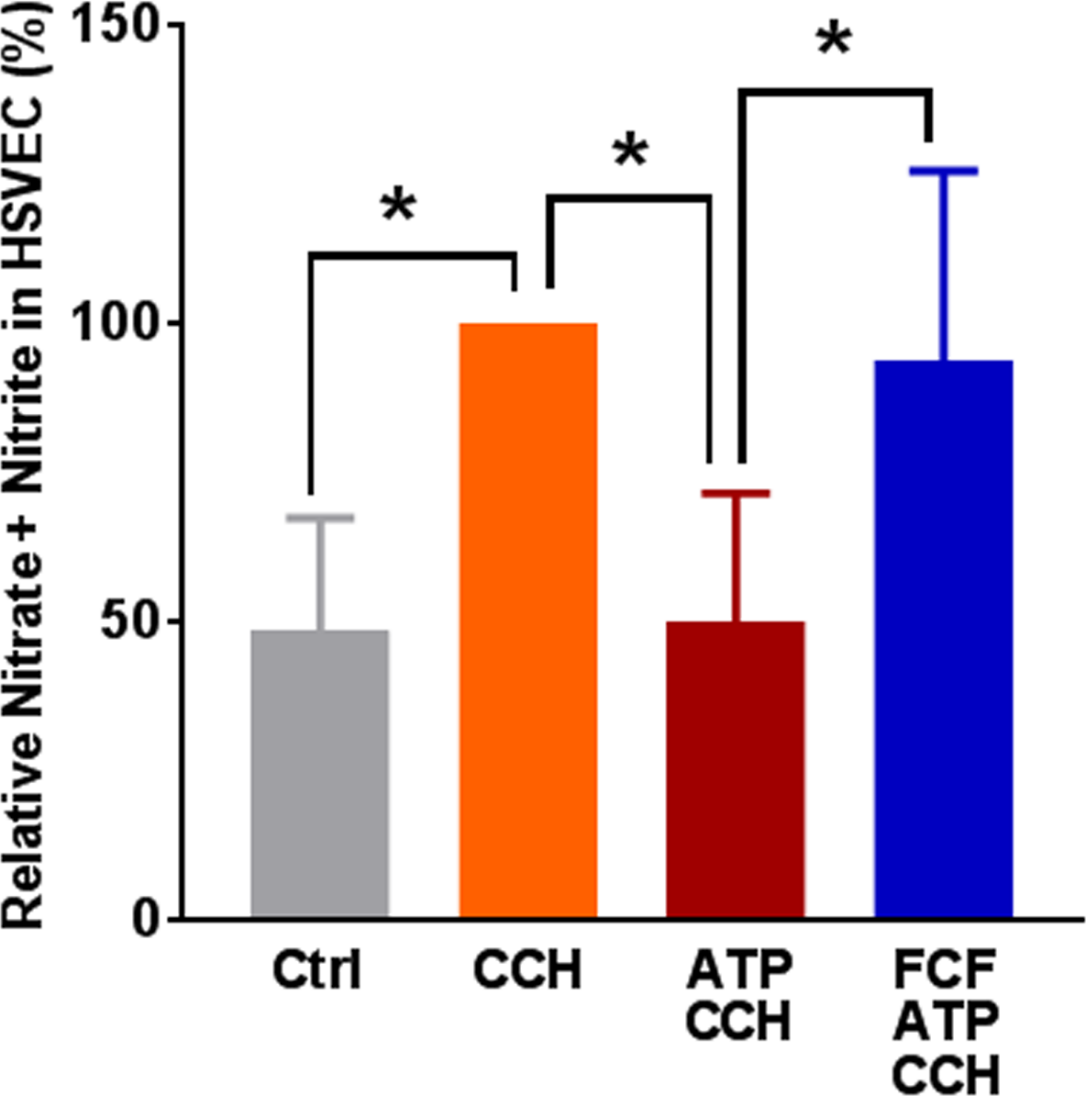
Treatment with eATP reduces nitric oxide production in HSV endothelial cells. HSVEC (>70% confluence, passages 2–4) were either untreated (Ctrl) or treated with ATP (2 mM) or ATP and FCF (100 μM) for 2 hours. Cells were stimulated with carbachol (1 μM, CCH) for 10 min at 37°C, and nitric oxide (NO) generated by the cells was measured as nitrite. NO generated with CCH was set as 100%, n = 4 passages, *p < 0.05, (One way ANOVA).

To focus specifically on endothelial cell responses, human saphenous vein endothelial cells (HSVEC) were used. Protein lysates from different passages (2–5) were examined for the expression of P2X7R by immunoblotting. A major band at 75 KDa was identified along with a minor band above 100KDa (S2 Fig). CCH stimulates nitric oxide (NO) production by endothelial cells, which leads to relaxation of the underlying vascular smooth muscle [28]. Stimulation of HSVEC with CCH led to increases in NO levels (49 ± 9% vs 100%, p < 0.05, Fig 3 and S3 Fig). To determine if eATP impairs endothelial-dependent relaxation by inhibiting NO production, HSVEC were treated with eATP and NO production was measured after CCH stimulation. eATP treatment inhibited CCH-stimulated NO production (50 ± 8% vs 100%, p < 0.05, Fig 3). Co-treatment with ATP and FCF prevented the eATP-induced decrease in NO production (50 ± 8% vs 94 ± 14%, p < 0.05, n = 4, Fig 3) suggesting that impaired CCH stimulated NO production after eATP treatment was due to P2X7R activation.

### eATP stimulation leads to increased phosphorylation of p38 MAPK

P2X7R activation in neural injury is associated with activation of p38 MAP kinase (p38 MAPK) [29–31]. To determine if eATP-induced activation of P2X7R in HSVEC leads to activation of p38 MAPK, HSVEC were treated with eATP or BzATP for different time points and phosphorylation of p38 MAPK was determined by western blotting. eATP induced a time-dependent phosphorylation of p38 MAPK with phosphorylation significantly increased by 10 minutes, and persistently elevated for 2 hours (S4 Fig). Treatment of HSVEC with BzATP (0.25 and 0.5 mM) also significantly increased phosphorylation of p38 MAPK (S5 Fig).

**Fig 4:**
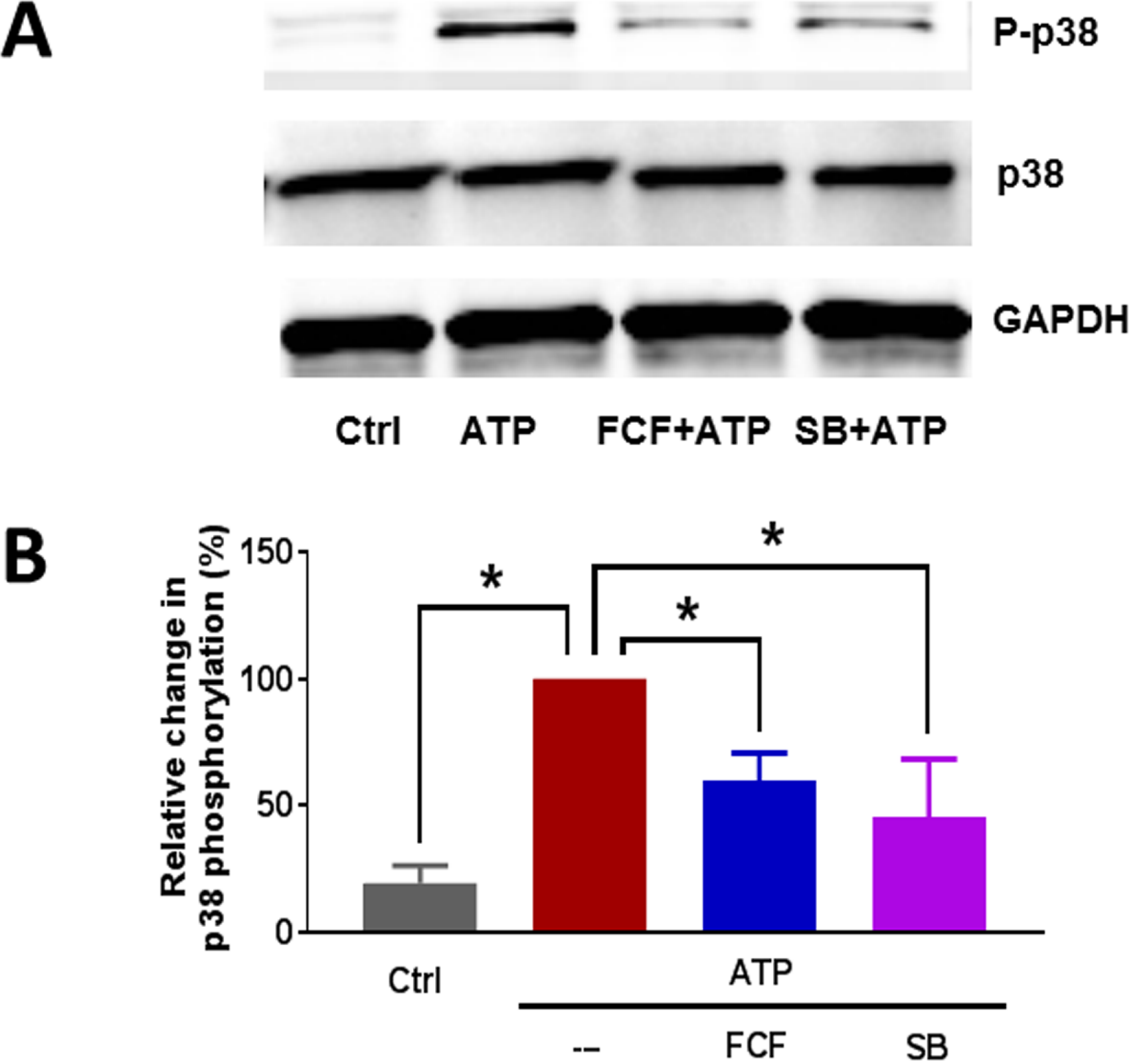
eATP induced phosphorylation of p38 MAPK in HSV endothelial cells. HSVEC were untreated (Ctrl), or treated with ATP (2 mM) for 30 minutes. Some cells were pre-treated with FCF (100 μM), or SB203580 (SB, 20 μM) for 1 hour. Phospho-p38 MAPK and total p38 MAPK proteins were quantitated with immunoblotting (adjusted to the loading control GAPDH). (**A**) Representative western blots of phospho-p38 MAPK and p38 MAPK. **(B**) Cumulative data showing the relative percent phosphorylation of p38 MAPK. Phosphorylation with eATP alone was set as 100%, n = 4 passages,* p < 0.05, (One way ANOVA).

To confirm that p38 MAPK activation was due to activation of P2X7R, HSVEC were pretreated with FCF. As a control, HSVEC were also pre-treated with the p38 MAPK inhibitor, SB 203580. The cells were then treated with eATP for 30 min and phosphorylation of p38 MAPK was determined. eATP-induced phosphorylation of p38 MAPK was inhibited by FCF (60 ± 5% vs 100%, p<0.05) and by the p38 MAPK inhibitor, SB 203580 (46 ± 11% vs 100%, p < 0.05, Fig 4).

**Fig 5:**
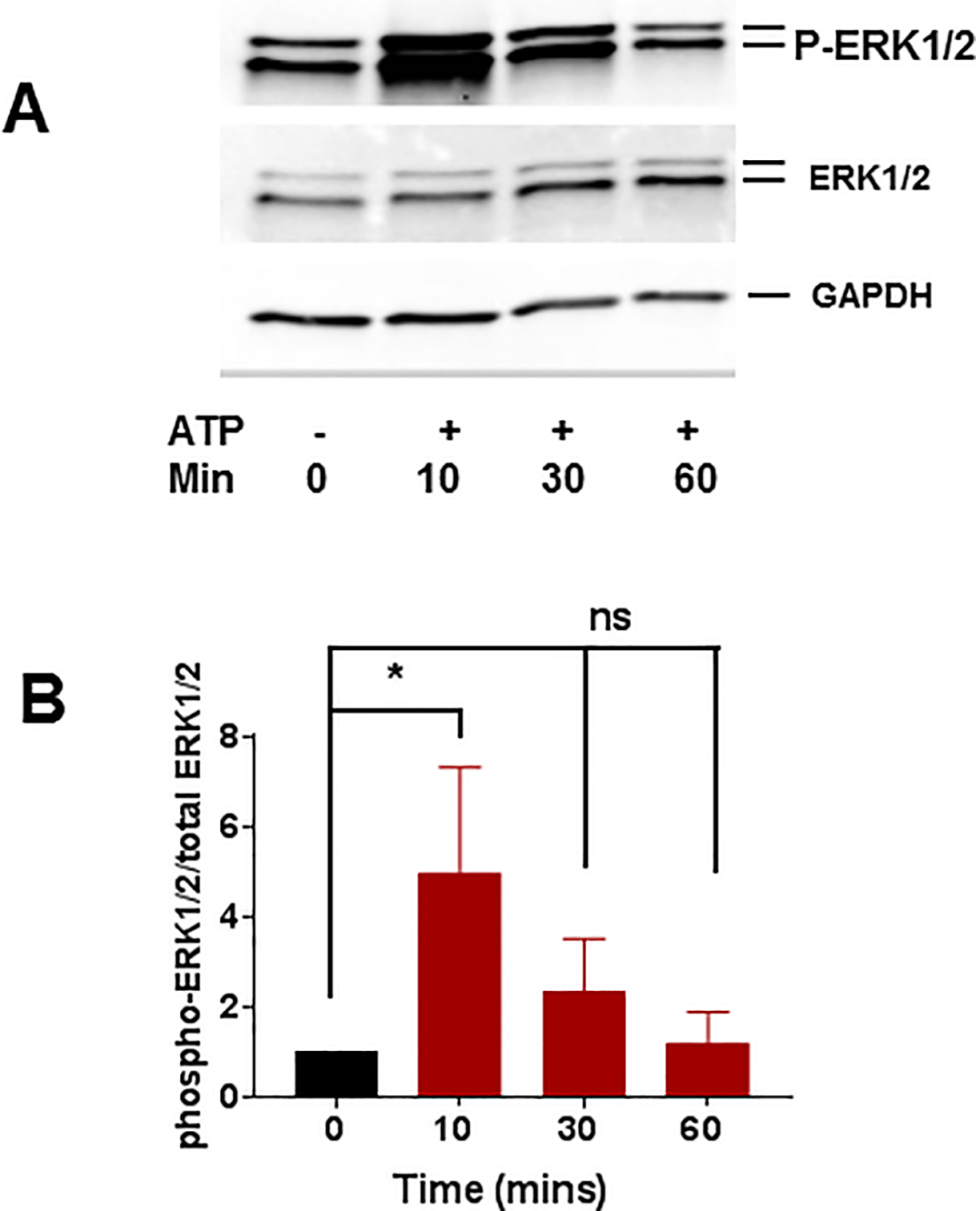
Time course of phosphorylation of p-ERK1/2 MAPK in ATP treated HSVEC. HSVEC were untreated (0) treated with ATP (2 mM) for various time points (10, 30, and 60 minutes) and phospho p44/42 MAPK (P-ERK1/2) and total p44/42 (ERK1/2) proteins were quantitated with immunoblotting (adjusted to the loading control GAPDH). (**A**) Representative western blot of phospho ERK1/2, **(B**) Cumulative data showing the phosphorylation (fold) of p ERK1/2 MAPK, * p < 0.05, N = 4, (One way ANOVA).

**Fig 6:**
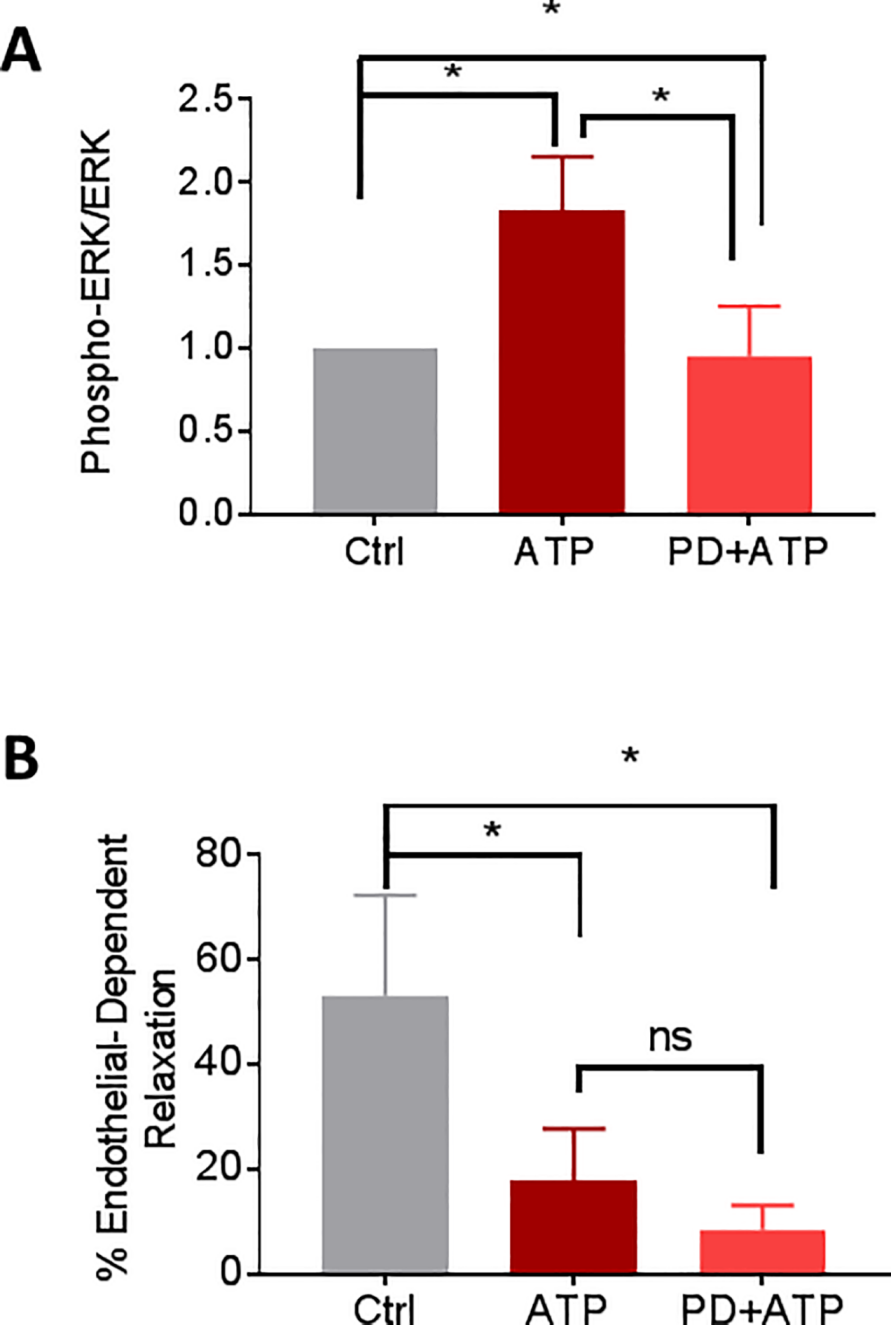
PD 98059 decreases the phosphorylation of ERK in HSVEC, but does not restore ATP-induced decrease in endothelial function in RA. **A.** HSVEC were untreated (Ctrl), treated with ATP (2 mM), or ATP with 10 μM PD 98059 (PD+ATP) for 20 min and phospho p44/42 MAPK (P-ERK1/2) and total p44/42 (ERK1/2) proteins were quantitated with immunoblotting (adjusted to the loading control GAPDH). Cumulative data showing the phosphorylation (fold) of p ERK1/2 MAPK, * p < 0.05, N = 3, paired t-test. **B.** RA rings were untreated (Ctrl), treated with eATP (20 mM, ATP) or ATP with 20 μM PD 98059 (PD+ATP) for 1hr, then suspended in the muscle bath and the vessels were pre-contracted with phenylephrine (5 x 10^−7^ M) and treated with carbachol (5 x 10^−7^ M). N = 5 segments from different rats, * p<0.05, ns = nonsignificant, (1 way ANOVA).

Activation of the other MAPK pathways such as the extracellular signal-regulated kinase (ERK) and c-JUN NH2-terminal protein kinase (JNK, also known as stress activated protein kinase, SAPK) in response to ATP treatment were also examined. HSVEC were treated with 2 mM ATP for different time points and activation of ERK and SAPK/JNK was determined by western blotting. ATP induced a transient increase in the phosphorylation of ERK1/2 at 10 min (1 vs 4.9±1.0 fold, Fig 5). Since ATP treatment increased the phosphorylation of ERK, the effect of ERK inhibitor, PD 98059 on ATP-induced phosphorylation was determined. PD 98059 decreased ATP-induced phosphorylation of ERK (Fig 6A and S6 Fig). To determine whether PD 98059 had an effect on restoring ATP-induced reduction of endothelial function, RA was treated with ATP (20 mM) or ATP with PD 98059 (20 μM) for 1 hr and endothelial-dependent relaxation was determined. There was no significant difference in the endothelial-dependent relaxation in ATP-treated RA in the presence of PD 98059 (17±4.5% vs 9±2% for ATP and PD+ATP, respectively, Fig 6B). There was no activation of SAPK/JNK in response to ATP in HSVEC (S7 Fig).

### eATP-induced reduction of nitric oxide production is inhibited by L-arginine and arginase inhibitor

**Fig 7:**
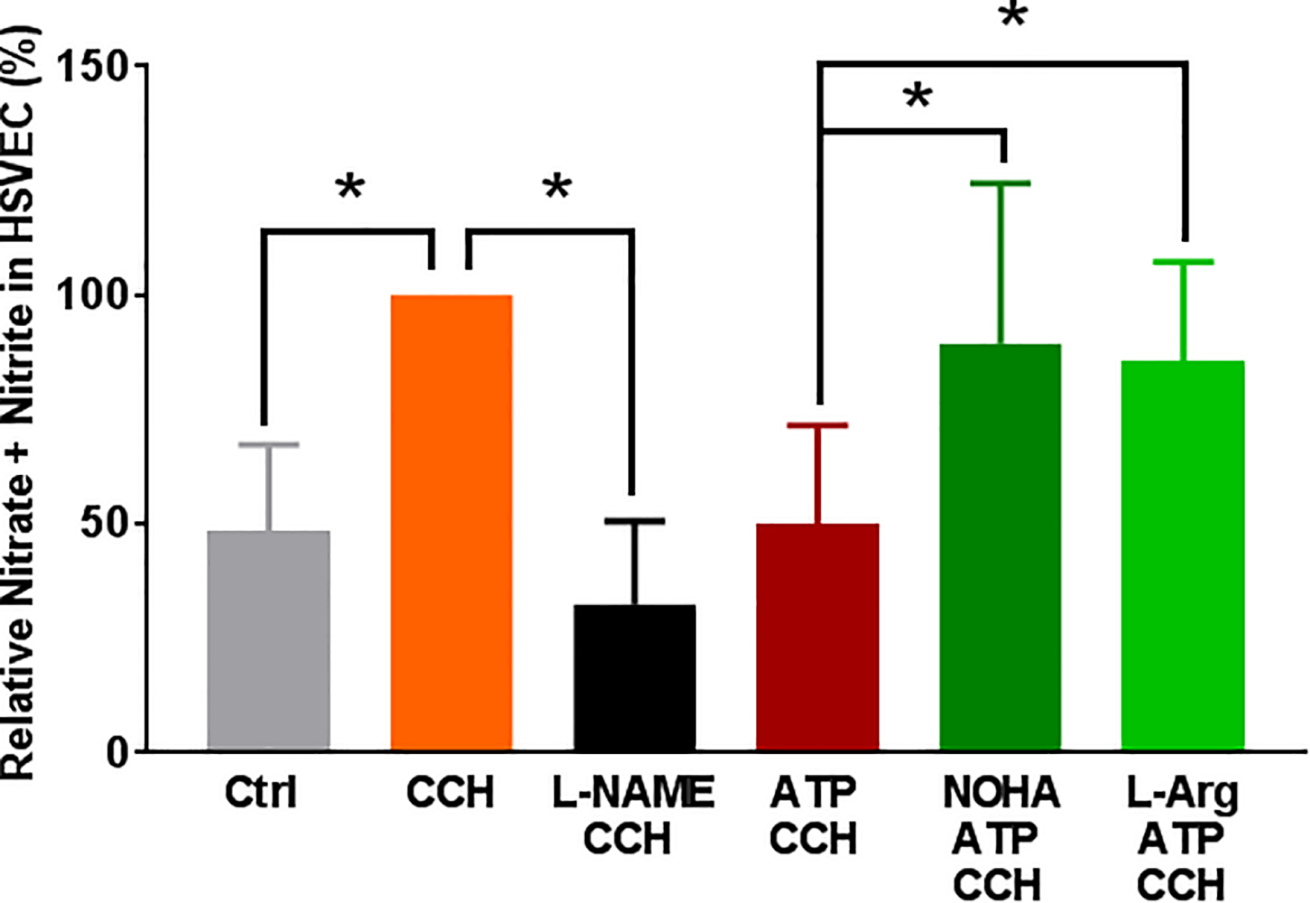
Effect of arginase inhibitor and L-arginine on eATP-induced reduction of nitric oxide production in HSV endothelial cells. HSVEC were either untreated (Ctrl), treated with ATP (2 mM), L-NAME, (100 μM), ATP with L-arginine (L-Arg, 2 mM), or ATP with NOHA (10 μM), for 2 hours. The cells were then stimulated with carbachol (CCH, 1 μM) for 10 minutes and the nitric oxide generated was measured as nitrite using the NO assay kit and relative percent of NO generated was calculated. NO generated with CCH was set as 100%, n = 6 passages, *p < 0.05, (One way ANOVA).

Since exogenous ATP and P2X7R/p38 MAPK activation lead to endothelial dysfunction by either decreased endothelial NO synthase (eNOS) activity or increased arginase activity, HSVEC were first treated with eATP and eNOS phosphorylation was determined. ATP treatment did not alter eNOS phosphorylation in HSVEC (S8 Fig). L-arginine is the substrate for NO production and arginase converts L-arginine into urea and L-ornithine, thus limiting NO substrate availability [32, 33] Since P38 MAPK activation has been associated with increased arginase activity [34, 35], we determined whether the effect of eATP could be reversed by treatment with an arginase inhibitor. When HSVEC were treated with eATP in the presence of the arginase inhibitor N^ω^ –hydroxy-nor-Arginine (NOHA) or L-arginine, ATP-induced decrease in CCH-stimulated NO production was prevented by NOHA (89 ± 15%) and L-arginine (86 ± 9%) when compared to ATP (50 ± 8%), p<0.05, Fig 7). Treatment of HSVEC with L-NG-Nitroarginine methyl ester (L-NAME, a non-hydrolyzable methyl ester of L-arginine that inhibits NOS), also inhibited CCH-induced NO production (33 ± 8% vs 100%, p < 0.05, Fig 7).

### Endothelial function was restored in stretch injured RA by inhibition of p38 MAPK and arginase

**Fig 8:**
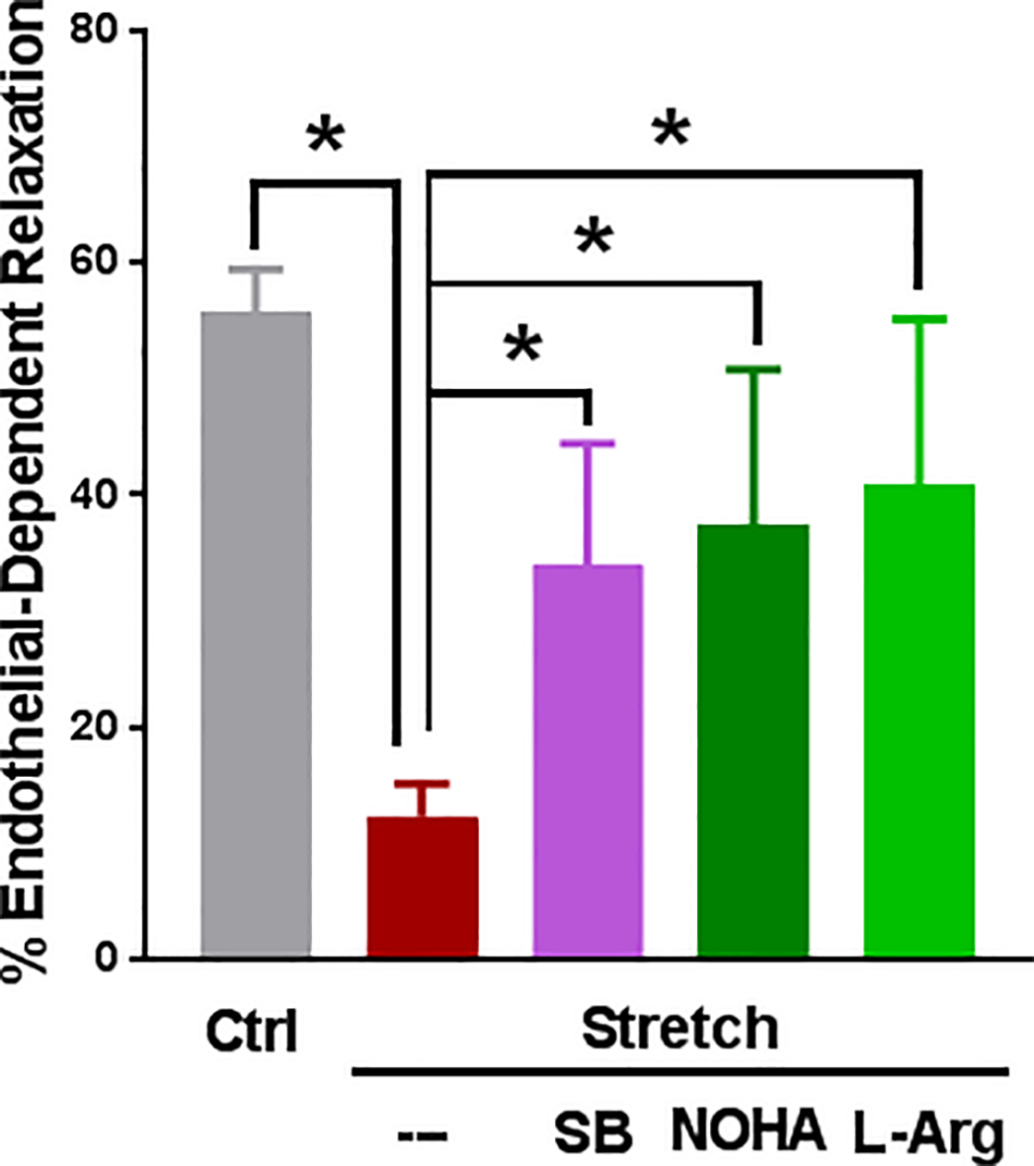
Stretch injury of RA decreased endothelial function that was restored with SB 203580. RA was unstretched (Ctrl), longitudinally stretched to twice its length (Stretch), or longitudinally stretched and treated with SB 203580 (SB, 20 μM), NOHA (50 μM) or L-Arginine (L-Arg, 2 mM) and endothelial relaxation was determined as described in Fig.1. n = 7 different rats, * p < 0.05, (paired t-test).

To determine whether impaired endothelial function after stretch injury-induced activation of P2X7R was due to activation of the p38 MAPK pathway and arginase activation in intact tissue, stretch-injured RA tissue was treated with SB 203580, NOHA, or L-arginine. Endothelial-dependent relaxation was restored after treatment with SB 203580 (34 ± 10% vs 15 ± 9%, p < 0.05), NOHA (37 ± 13%, vs 15 ± 9%, p<0.05), and L-arginine (41 ± 14%, vs 15 ± 9%, p < 0.05, Fig 8).

## Discussion

Because a functional endothelium is important for viable vascular function [9], reducing injury to the endothelial layer during harvest and preparation of HSV represents a plausible intervention to prevent vein graft failure and subsequent morbidity (myocardial infarction, limb loss, and repeat interventions) and mortality associated with graft failure.

Surgically-induced injury associated with vein graft preparation has been well described and straightforward methods to mitigate this injury have been developed [3–7, 11–15]. Surgery by its very nature leads to tissue injury and in the case of harvesting HSV, significant mechanical (traction) injury ensues. To characterize surgical vascular stretch injury, a model of subfailure overstretch of a reliable and reproducible vascular tissue, rat aorta, was developed [11]. Stretch injury leads to impaired vascular smooth muscle contraction [11]. In the current study, this model was used to demonstrate that subfailure overstretch injury also leads to impaired endothelial-dependent relaxation (Fig 1). Stretch-induced impaired endothelial-dependent relaxation was *restored* by treatment with P2X7R inhibitors, FCF and A740003, or by hydrolyzing ATP with apyrase after stretch injury (Fig 1A and 1B). Previous studies have demonstrated that P2X7R inhibitor A740003 restored stretch-induced reduction in contractile response in RA [19], reduces inflammation in human atherosclerotic vessels [26], and is effective in animal models of neuropathic and inflammatory pain [25]. Since HSV is typically stored in solution after harvest and prior to implantation, direct pharmacologic treatment of the graft during storage represents an opportunity for therapeutic intervention prior to implantation. Of note, FCF has been recently approved for use in marking HSV to maintain orientation after implantation [8].

Impaired endothelial-dependent relaxation was recapitulated by treatment of RA with exogenous ATP (Fig 2A), suggesting that ATP may be the molecular mediator of impaired endothelial function after stretch injury ATP is a multifunctional nucleotide that serves as an energy source, a component of RNA, and a substrate for intracellular signaling. ATP is normally retained within the cytoplasm and a steep ATP concentration gradient exists between the cytoplasm (10^−3^ to10^-2^ M) [27] and the extracellular space (10^−9^ to 10^−8^ M) [36]. Thus, tissue damage would lead to rapid release of high local concentrations of ATP consistent with the concentrations of eATP used in this study [37, 38].We have demonstrated earlier that stretch injury is associated with ATP release, P2X7R activation, and decreased contractile function in RA [20].

P2X7R are activated by ATP (EC_50_ of 300–800μM) [39] and are unusual in that **ATP stimulation of P2X7R leads to ATP release from P2X7R** [40, 41]. With sustained stimulation of the P2X7R with ATP, the receptor forms large pores that can lead to cytolysis and additional release of ATP [42]. P2X7R activation also leads to increased P2X7R expression [38]. P2X7R were expressed in the HSVEC (passages 3–5) that were used in this study (band ~75 KDa, S2 Fig) consistent with other studies demonstrating P2X7R expression [26, 43–47]. Previous studies have also identified P2X7R in the endothelial and medial layers of RA [19] suggesting that stretch-mediated release of ATP can activate P2X7R resulting in further release of ATP and decreased endothelial function. Endothelial cells from different sources have been shown to express different purinergic receptors including P2X1,3–7 and P2Y [43, 47, 48] and it is possible that ATP may activate other purinergic receptors along with P2X7R. However, treatment of RA with P2Y11 inhibitor did not restore ATP-induced reduction of endothelial-dependent relaxation (data not shown). Treatment of RA with BzATP, a selective agonist of P2X7R, dose dependently decreased endothelial-dependent relaxation confirming the role of P2X7R in the ATP mediated endothelial dysfunction (Fig 2B).

Finally, P2X7R activation is a potent inflammatory stimulus [49, 50]. Thus, ATP activation of the P2X7R could potentiate and modulate the response to injury by leading to further ATP release and activation of a sustained inflammatory response [18, 51].

ATP-mediated inhibition of NO release (Fig 3) was associated with activation of p38 MAPK in HSVEC (Fig 4). P38 MAPK activation has been associated with increased arginase activity [34, 35], the hydrolytic enzyme responsible for conversion of L-arginine, thus limiting substrate availability for eNOS. Treatment of cells with arginase inhibitor, or adding L-arginine [52], prevented ATP-induced inhibition of NO production (Fig 7). Several arginase inhibitors have been developed for treatment of vascular diseases [53]. Extracellular L-arginine has been shown to restore endothelial function in isolated vessels and systemically in settings of endothelial dysfunction [54]. L-arginine is a component of an endothelial preservation solution (DuraGraft) under development for storage of HSV after harvest and prior to implantation [55]. Effect of ATP treatment on other MAP kinases demonstrated a transient increase in the phosphorylation of ERK1/2 in HSVEC (Fig 5) which was inhibited by the ERK inhibitor PD 98509 (Fig 6A). However, treatment of RA with ERK inhibitor did not restore ATP-induced inhibition of endothelial-dependent relaxation (Fig 6B), suggesting that while activation of ERK pathways are associated with ATP-induced activation of P2X7R, there is no corresponding physiologic effect. Muscle bath studies with intact tissues provides a unique opportunity to correlate physiologic responses with signaling events. There was no increase in the phosphorylation of JNK in response to ATP in HSVEC (S7 Fig).

A growing list of P2X7R antagonists have been used successfully *in vitro* and in animal studies [18, 56]. Antagonists to the P2X7R ameliorate cascades initiated by injury in the lung, kidney, and nervous system [38, 50, 57]. Most P2X7R antagonists are in development to treat neurologic disorders and pain [58]. Two completed clinical studies demonstrated a clean safety profile of P2X7R inhibitors [59, 60]. Targeting the P2X7R to treat endothelial injury during vascular intervention procedures represents a new field for the use of P2X7R antagonists.

Limitations of this study include the use of an arterial tissue model (RA). The RA stretch model is a model of **pathologic injury** and not physiologic mechanical forces such as those that occur with arterialization of the graft after implantation. In addition, the subfailure overstretch RA model represents a well characterized, inexpensive, high throughput model and while injury of both HSV and PSV [4, 6, 12] leads to impaired vasomotor function that is restored by FCF, further work is needed to extrapolate the findings in the RA model to these and other tissues. P2X7R activation has been shown to lead to increased P2X7R expression [38], apoptosis, and an inflammatory response [16] in other cellular systems, but the longer term consequences of P2X7R activation in vascular tissues remains to be determined. Finally, P2X7R may also potentiate the response to injury due to further ATP release from the receptor itself [42] and increased receptor expression [38], and the kinetics of ATP release after injury remain to be delineated. However, data from these other systems suggest that P2X7R activation after stretch injury may not only modulate but also **potentiate** the response to injury.

**Fig 9:**
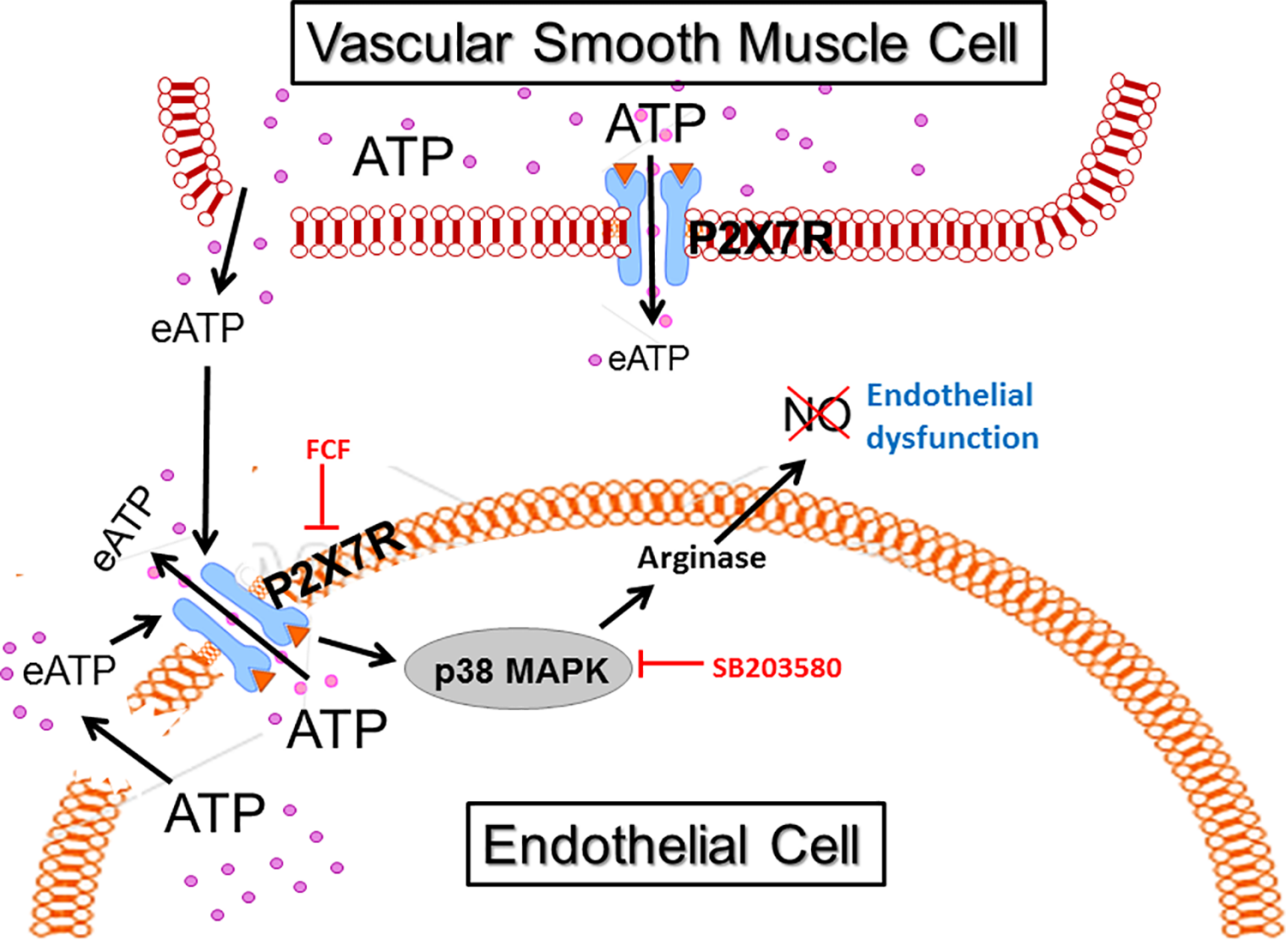
Harvest traction stretch injury led to ATP release, activation of P2X7R and endothelial dysfunction. During endoscopic harvest of human saphenous vein (HSV), traction stretch injury leads to release of ATP from vascular cells. The high concentrations of extracellular ATP (eATP) activate P2X7 receptors (P2X7R) which leads to further release of ATP from P2X7R channels and activation of p38MAPK. Brilliant blue FCF (FCF) is an inhibitor of P2X7R and SB203580 is a small molecule inhibitor of p38 MAPK. P38 MAPK leads to arginase activation which decreases nitric oxide (NO) resulting in endothelial dysfunction.

Taken together, these data suggest that stretch injury during surgical harvest leads to release of ATP, activation of P2X7R/p38 MAPK, and inhibition of NO production by activation of arginase (Fig 9), which results in impaired endothelial function. Treatment of vascular tissues with P2X7R inhibitors, such as FCF, arginase inhibitors such as NOHA, and modifying the downstream arginase activation by increasing the availability of the NO substrate, L-arginine, represent available approaches to ameliorate stretch injury after the injury has occurred and prior to implantation as bypass conduits. Modifying current surgical harvest and preparation with simple and straightforward techniques, in conjunction with development of pharmacologic approaches to limit molecular events that modulate injury and amplification of the injury response, would improve conduit function and likely improve vein graft durability.

## Supporting information

S1 Fig Extracellular ATP stimulation of RA decreased endothelial function. RA rings were suspended in the muscle bath and the vessels were treated with eATP for 1hr, and were pre-contracted with phenylephrine (5 x 10^−7^ M) and treated with carbachol (5 x 10^−7^ M). Representative tracing of control (Ctrl, A) compared to ATP-treated rings (ATP, B).
(TIF)

S2 Fig Expression of P2X7R in human saphenous vein endothelial cells (EC) and HEK 293 cells (HEK). HSVECS (>70% confluence, passages 2–4) or HEK cells were lysed and proteins were separated by SDS PAGE and transferred to nitrocellulose membrane and probed with **A,** anti P2X7 R antibodies preabsorbed with peptide (preabsorbed) or **B**, anti P2X7 R antibodies (P2X7 R). Representative western blot of P2X7 R, N = 4 different passages.
(TIF)

S3 Fig ATP treatment reduces nitric oxide production in HSVEC. HSVEC were either untreated or treated with 2 mM ATP for 2 hr and were stimulated with 1μM carbachol for 10 min at 37°C. Cells were scraped in cold assay buffer and nitric oxide generated was measured as nitrate using the kit (Abcam). * Significant compared to untreated CCH stimulated, n = 4, p<0.05,(paired t-test).
(TIF)

S4 Fig Time course of phosphorylation of p38 MAPK in ATP treated HSVEC. HSVEC were untreated (0), treated with ATP (2 mM) for various time points (10, 30, 60 and 120 minutes) and phospho p38 MAPK (P-p38) and total p38 (p38) proteins were quantitated with immunoblotting (adjusted to the loading control GAPDH). (**A**) Representative western blots of phospho p38MAPK, and p38 MAPK, **(B**) Cumulative data showing the relative fold phosphorylation of p38 MAPK with respect to zero time point, n = 4–5 passages, * p < 0.05,.(paired t-test).
(TIF)

S5 Fig Activation of p38 MAPK with BzATP in HSVEC. HSVEC were either untreated (control) or treated with BzATP (0.25 mM and 0.5mM) in 50% growth medium diluted with basal medium for 2 h. Proteins were extracted and separated on 4–20% criterion gels and transferred to nitrocellulose. Phospho p38 and p38 proteins were identified by western blot analysis using antibodies to phospho p38 and total p38 (Cell signaling). n = 3, * p<0.05 between control and 0.5 mM BzATP, ns = not significant, (t-test).
(TIF)

S6 Fig PD 98059 inhibits ATP-induced phosphorylation of ERK in HSVEC. HSVEC were untreated (Ctrl), treated with ATP (2 mM) or ATP with PD 98059 (10 μM, PD+ATP) 20 minutes) and phospho p44/42 MAPK (P-ERK1/2) and total p44/42 (ERK1/2) proteins were quantitated with immunoblotting (adjusted to the loading control GAPDH). Representative western blot, N = 3.
(TIF)

S7 Fig Time course of phosphorylation of p-SAPK/JNK in ATP treated HSVEC. HSVEC were untreated (0) treated with ATP (2 mM) for various time points (10, 30, and 60 minutes) and phospho SAPK/JNK (P-p54/p46 SAPK/JNK) and total JNK (SAPK/JNK) proteins were quantitated with immunoblotting (adjusted to the loading control GAPDH). Representative western blot, N = 3.
(TIF)

S8 Fig ATP stimulation did not significantly change eNOS phosphorylation in HSVEC. HSVEC were either untreated (Ctrl), or treated with ATP (1 and 2 mM) for 2hour. Phospho eNOS and total eNOS were identified by western blot analysis using antibodies to phospho eNOS and total eNOS and adjusted to the loading control GAPDH. n = 4 passages, ns, not significant, * p >0.05, for 1 mM and 2 mM, respectively, (t-test).
(TIF)
