# Supplementary figures and images for: Vascular surgical stretch injury leads to activation of P2X7 receptors and impaired endothelial function
Source: PLoS One. 2017 Nov 14;12(11):e0188069. doi: 10.1371/journal.pone.0188069 (PMC5685620; doi:10.1371/journal.pone.0188069)

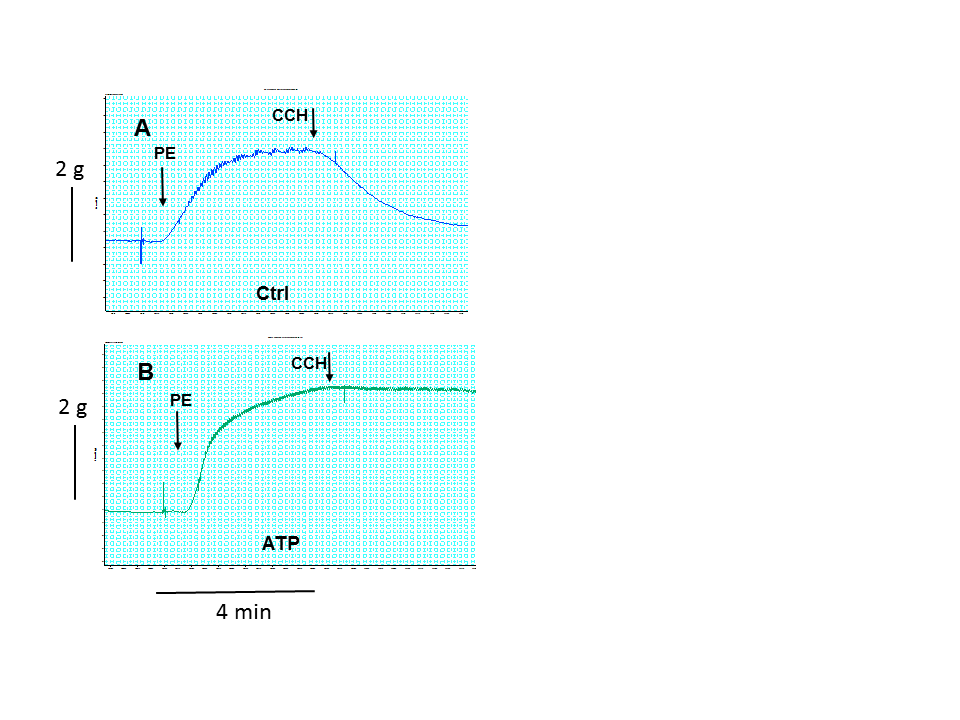

Supplement: S1 Fig — RA rings were suspended in the muscle bath and the vessels were treated with eATP for 1hr, and were pre-contracted with phenylephrine (5 x 10^−7^ M) and treated with carbachol (5 x 10^−7^ M). Representative tracing of control (Ctrl, A) compared to ATP-treated rings (ATP, B). (TIF) [file pone.0188069.s001.tif]

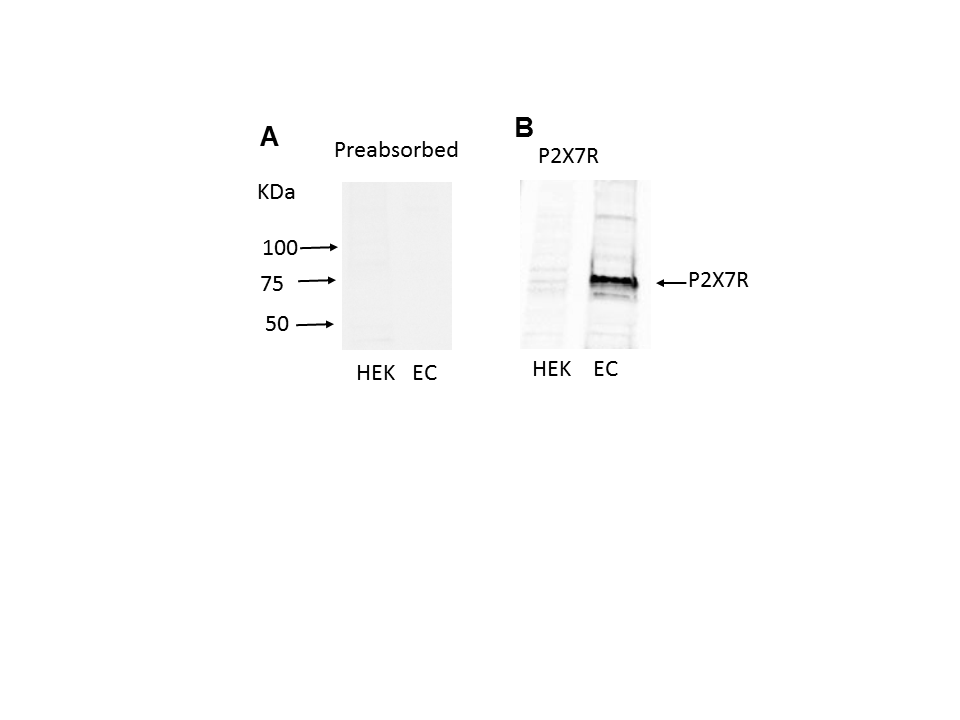

Supplement: S2 Fig — HSVECS (>70% confluence, passages 2–4) or HEK cells were lysed and proteins were separated by SDS PAGE and transferred to nitrocellulose membrane and probed with A, anti P2X7 R antibodies preabsorbed with peptide (preabsorbed) or B, anti P2X7 R antibodies (P2X7 R). Representative western blot of P2X7 R, N = 4 different passages. (TIF) [file pone.0188069.s002.TIF]

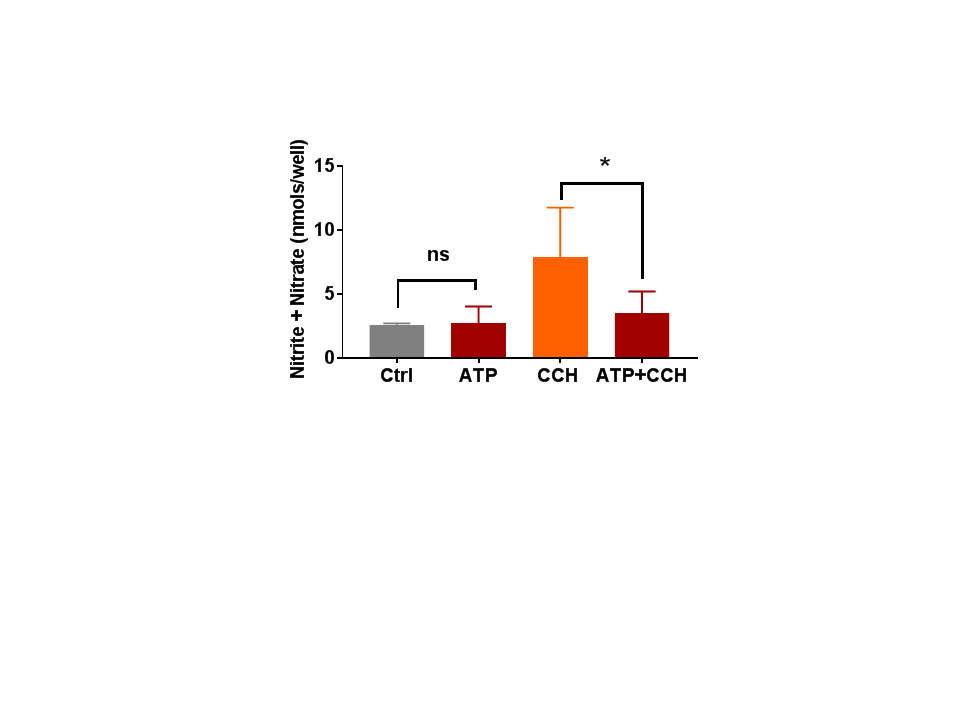

Supplement: S3 Fig — HSVEC were either untreated or treated with 2 mM ATP for 2 hr and were stimulated with 1μM carbachol for 10 min at 37°C. Cells were scraped in cold assay buffer and nitric oxide generated was measured as nitrate using the kit (Abcam). * Significant compared to untreated CCH stimulated, n = 4, p<0.05,(paired t-test). (TIF) [file pone.0188069.s003.tif]

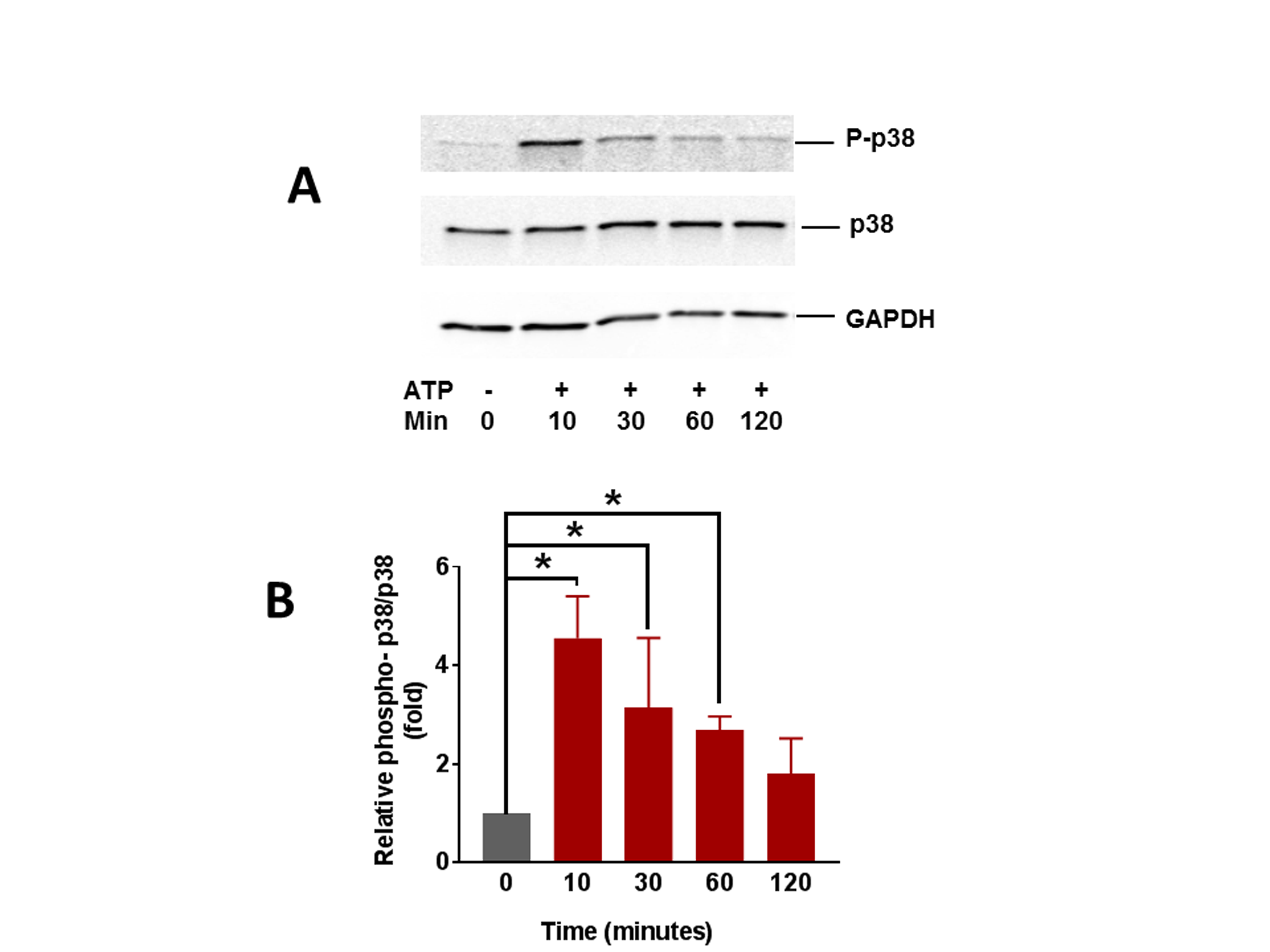

Supplement: S4 Fig — HSVEC were untreated (0), treated with ATP (2 mM) for various time points (10, 30, 60 and 120 minutes) and phospho p38 MAPK (P-p38) and total p38 (p38) proteins were quantitated with immunoblotting (adjusted to the loading control GAPDH). (A) Representative western blots of phospho p38MAPK, and p38 MAPK, (B) Cumulative data showing the relative fold phosphorylation of p38 MAPK with respect to zero time point, n = 4–5 passages, * p < 0.05,.(paired t-test). (TIF) [file pone.0188069.s004.TIF]

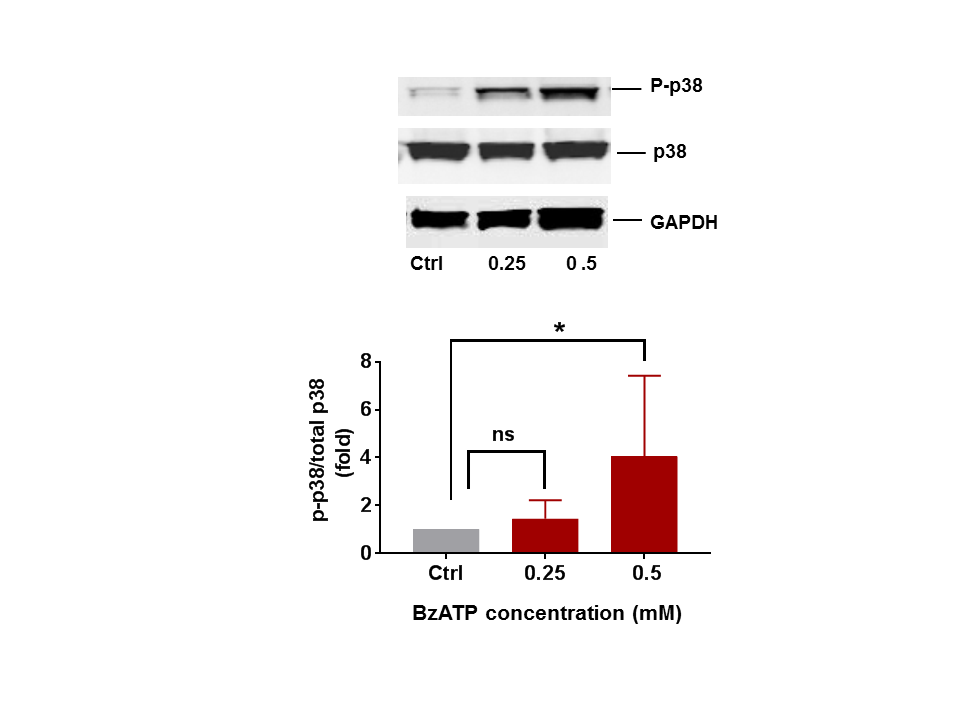

Supplement: S5 Fig — HSVEC were either untreated (control) or treated with BzATP (0.25 mM and 0.5mM) in 50% growth medium diluted with basal medium for 2 h. Proteins were extracted and separated on 4–20% criterion gels and transferred to nitrocellulose. Phospho p38 and p38 proteins were identified by western blot analysis using antibodies to phospho p38 and total p38 (Cell signaling). n = 3, * p<0.05 between control and 0.5 mM BzATP, ns = not significant, (t-test). (TIF) [file pone.0188069.s005.tif]

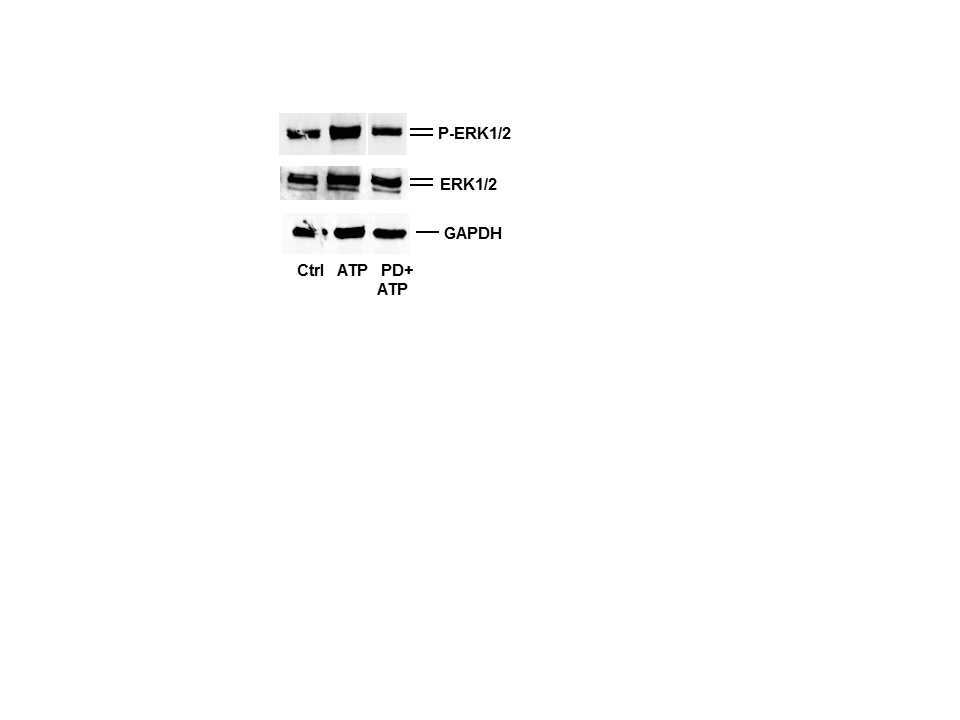

Supplement: S6 Fig — HSVEC were untreated (Ctrl), treated with ATP (2 mM) or ATP with PD 98059 (10 μM, PD+ATP) 20 minutes) and phospho p44/42 MAPK (P-ERK1/2) and total p44/42 (ERK1/2) proteins were quantitated with immunoblotting (adjusted to the loading control GAPDH). Representative western blot, N = 3. (TIF) [file pone.0188069.s006.TIF]

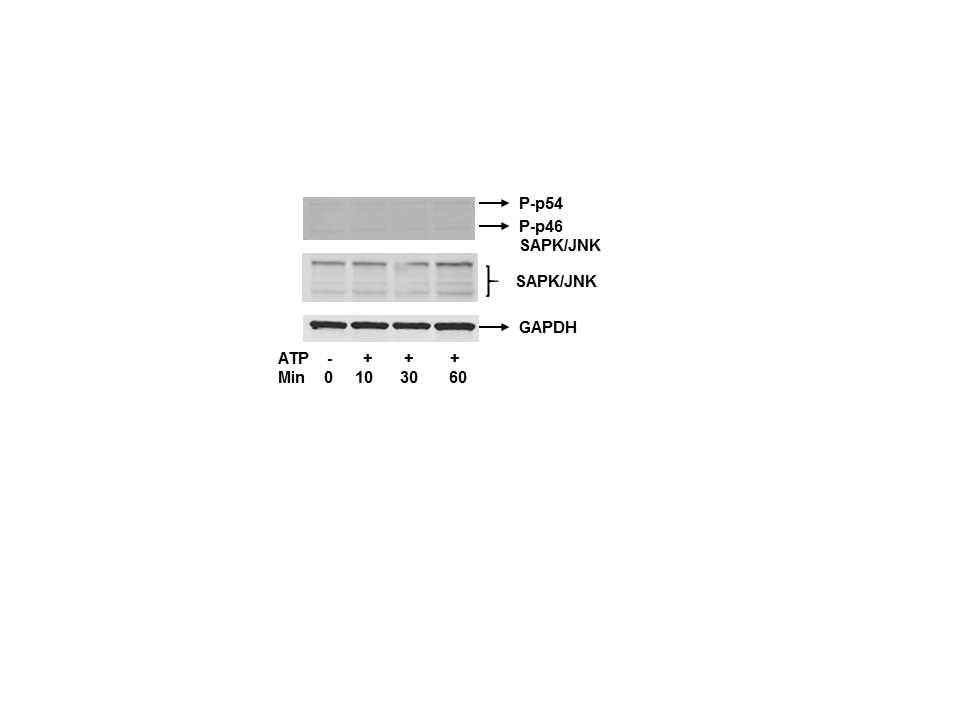

Supplement: S7 Fig — HSVEC were untreated (0) treated with ATP (2 mM) for various time points (10, 30, and 60 minutes) and phospho SAPK/JNK (P-p54/p46 SAPK/JNK) and total JNK (SAPK/JNK) proteins were quantitated with immunoblotting (adjusted to the loading control GAPDH). Representative western blot, N = 3. (TIF) [file pone.0188069.s007.TIF]

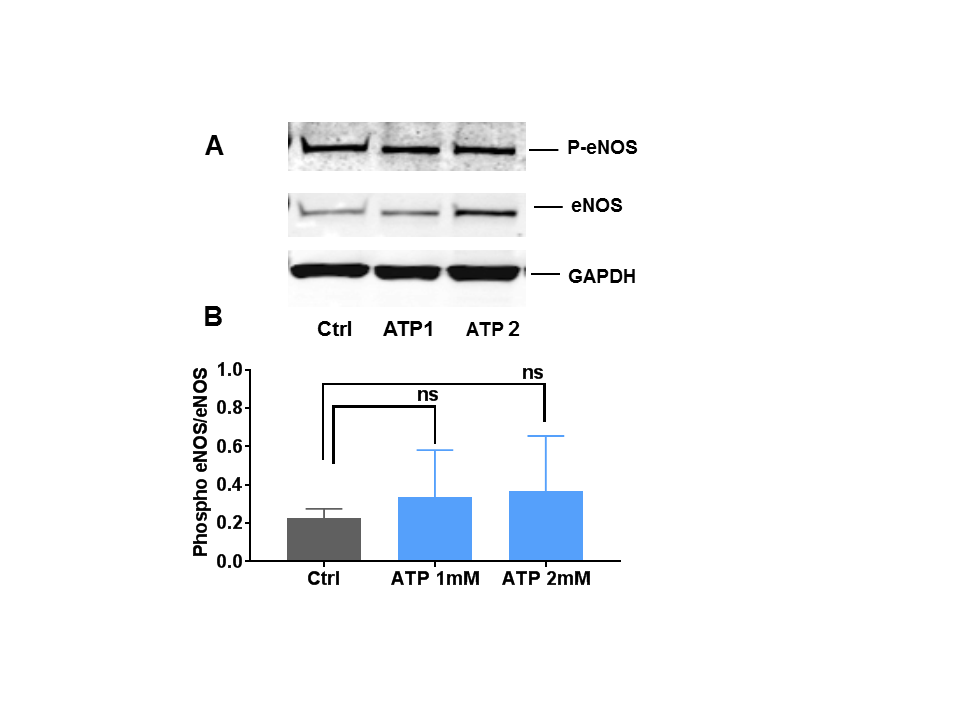

Supplement: S8 Fig — HSVEC were either untreated (Ctrl), or treated with ATP (1 and 2 mM) for 2hour. Phospho eNOS and total eNOS were identified by western blot analysis using antibodies to phospho eNOS and total eNOS and adjusted to the loading control GAPDH. n = 4 passages, ns, not significant, * p >0.05, for 1 mM and 2 mM, respectively, (t-test). (TIF) [file pone.0188069.s008.TIF]
